# Detection of the circulating antigen 14-3-3 protein of *Schistosoma japonicum *by time-resolved fluoroimmunoassay in rabbits

**DOI:** 10.1186/1756-3305-4-95

**Published:** 2011-05-28

**Authors:** Chun-Yan Qian, Biao Huang, Chuan-Xin Yu, Jue Zhang, Xu-Ren Yin, Jie Wang, Li-Jun Song, Wei Zhang, Xue-Dan Ke

**Affiliations:** 1 Jiangsu Institute of Parasitic Diseases, Wuxi, Jiangsu Province 214064, China; 2 Jiangsu Institute of Nuclear Medicine, Wuxi, Jiangsu Province 214063, China

## Abstract

**Background:**

Schistosomiasis remains a major public health concern that afflicts millions of people worldwide. Low levels of *Schistosoma *infection require more sensitive diagnostic methods. In this study, a time-resolved fluoroimmunoassay (TRFIA) was developed for detecting the signal transduction protein 14-3-3, a circulating antigen of *Schistosoma japonicum*.

**Results:**

The detection limit of 14-3-3-TRFIA was 0.78 ng/ml, with a linear measurement range from 0.78 to 800 ng/ml. The average intra-assay and inter-assay variability of this TRFIA was 8.9% and 12.2% respectively, and the mean recovery rate ranged from 92.1% to 115.5%. Within the first 21 days post-infection in rabbits, the positive rates of the 14-3-3-TRFIA were distinctly higher compared to ELISA. All these findings illustrate that 14-3-3-TRFIA has a higher detection efficacy and is a good early diagnostic method for active *Schistosoma *infection.

**Conclusions:**

A sandwich TRFIA for detecting the circulating antigen 14-3-3 of *S. japonicum *has been developed, and has demonstrated to be a good potential diagnostic method for schistosomiasis.

## Findings

Over the past 50 years, the ongoing national control program has made great progress in controlling schistosomiasis japonica in China, but this disease is still a major public health concern that afflicts millions of people in endemic areas [1,2]. Definite diagnosis of the disease plays a key role in the control of schistosomiasis [3]. Detection of *Schistosoma *circulating antigen is an effective approach to discriminate between previous exposure and current infection [3]. Effective chemotherapy and other interventions, such as local environment alternation [4], livestock in pens, and health education, have dramatically reduced schistosome infections, and major infection in endemic areas remains on a low level [5-7]. If the level of circulating antigen in host serum is less than the detection limit of a diagnostic method, false-negative results will be obtained, which would result in some patients missing treatment. Selecting an abundant circulating antigen as target would be very helpful for developing a highly sensitive diagnostic method of schistosomiasis. The signal transduction protein 14-3-3 of *S. japonicum *is abundant in excretory-secretary extracts [8], soluble egg extracts [9] and adult worm extracts [10], and can be used for the diagnosis of acute and chronic *S. japonicum *infections [11]. In order to further improve the detection sensitivity of 14-3-3, a sandwich time-resolved fluoroimmunoassay (TRFIA) was developed using a pair of monoclonal antibodies, and it could be shown that TRFIA has a higher sensitivity in detecting 14-3-3 antigen of *S. japonicum *than an enzyme-linked immunosorbent assay (ELISA).

Diethylenetriaminepentaacetate (DTPA), bovine serum albumin (BSA), Tris and Triton X-100 were purchased from Sigma (St. Louis, MO, USA). A PD-10 column and a sepharose CL-6B column were obtained from the Pharmacia Company (Chalfont St Giles, UK). Pure water was produced by Barnstead Equipment (Dubuque, Iowa, USA). Flat-bottomed 96-well polystyrene microtiter plates were purchased from Nunc International (Roskilde, Denmark). Eu-labeling reagent 1244-302, including N'-[p-isothiocyanatobenzyl]-diethylenetriamine-N1, N2, N3, N4-tetraacetic acid, was obtained from Perkin-Elmer (Waltham, Massachusetts, USA). Beta-NTA was synthesized in our laboratory. AutoDELFIA_1235 _(Perkin-Elmer, Waltham, Massachusetts, USA) was used to measure Eu^3+ ^fluorescence in microtiter wells. An ELISA reader was purchased from Tecan Sunrise, Switzerland. All other reagents used were of analytical grade.

*S. japonicum *cercariae (Chinese strain), freshly released from infected intermediate host snails (*Oncomelania hupensis*), were provided by the Department of Snail Biology, Jiangsu Institute of Parasitic Diseases, China.

Twelve young Japanese rabbits, each weighing about 2.5 kg, were purchased from the Experimental Animal Facility of Nanjing General Hospital of Nanjing Military Command, China, and raised in the Department of Animal Experiment, Jiangsu Institute of Parasitic Diseases. All rabbits were randomly divided into Group A and Group B. Group A included 10 rabbits, each infected with 500 cercariae of *S. japonicum *by abdominal skin without any treatment. Group B included two rabbits, and was used as negative control without any infection or treatment. Serum samples were collected at 0, 7, 14, 21, 28 days post-infection from all rabbits and stored at -80°C for subsequent experiments. All rabbits were sacrificed at 42 days post-infection. Their worm burden and egg burden were measured. All experiments conformed to local government regulations and Chinese national laws on animal ethics.

Two monoclonal antibodies (McAbs), 5C6 and 5D1, against recombinant signal transduction protein 14-3-3 of *S. japonicum *were prepared as described previously [12,13].

McAb 5C6 was labeled according to previously published methods [14,15]. The recombinant plasmid expressing the fusion protein of glutathione-S-transferase and signal transduction protein 14-3-3 was constructed and purified [16]. The 14-3-3 calibrators were prepared by serially diluting purified 14-3-3 protein in assay buffer, ranging from 0 - 800 ng/ml [50 mmol/l Tris-HCl (pH 7.8), 0.9% NaC1, 0.2% BSA, 0.05% NaN_3_, 20 μmol/l DTPA, and 0.1% Tween-20].

After optimizing the test conditions, the following TRFIA procedure was adopted: McAb 5D1 was dissolved in coating buffer (0.05 M sodium bicarbonate buffer, pH 9.6) at a concentration of 10 μg/ml. One hundred μl of this solution was added to each well and the plate was incubated overnight at 4°C. After removing the antibody solution, the plate was blocked with 20 g/l BSA (200 μl/well) in assay buffer for 2 h at room temperature. After the blocking solution was removed, the plates were dried in a high vacuum, and then stored at -20°C in a sealed plastic bag with desiccant.

The procedure for 14-3-3-TRFIA was performed by using a two-step and non-competitive "sandwich-type" protocol. Briefly, 100 μl of calibrators or serum samples without dilution were added into the coated wells, and then the plate was incubated with continuous shaking at 37°C for 2 h. After four washes in washing solution [50 mmol/l Tris-HCl buffer (pH 7.8), containing 0.9% NaCl, 0.2% Tween-20, and 0.05% NaN_3_], 100 μl of Eu^3+^-5C6 conjugate diluted in assay buffer was added into each well. The plate was incubated at 37°C for 2 h. After six washes, 200 μl of enhancement solution was added into each well. The plate was shaken for 5 min before fluorescence reading. All analysis was performed by AutoDELFIA_1235 _software. Duplicates of each sample were run. The calibration curve was plotted and concentrations in unknown samples were measured using the Multicalc software program, employing a spine algorithm on logarithmically transformed data. The antigen levels in sera were expressed as response counts. Response counts of samples above 2.1 times of the negative controls were judged as positive.

After optimizing the assay conditions, the following ELISA procedure was adopted: McAb 5D1 was dissolved in coating buffer at a concentration of 5 μg/ml. One hundred μl of this solution was added to each well and the plate was incubated overnight at 4°C. After removing the antigen solution, the plate was blocked by adding 200 μl/well blocking solution [5% skimmed milk power in 0.02 M phosphate buffered saline with 0.05% Tween-20 (PBST), pH 7.2] at 37°C for 1 h. After the wells were emptied, 100 μl of serum sample without dilution or calibrator was added to per well and incubated at 37°C for 1 h. After four washes, 100 μl of horseradish peroxidase (HRP)-conjugated 5C6 solution diluted in PBST was added and incubated at 37°C for 1 h. The plate was washed as above and the reaction was visualized by adding 100 μl of TMB substrate solution (GenScript, China) to each well for 5 - 10 min in dark at room temperature. The reaction was stopped by adding 100 μl/well of stop solution (1 M H_2_SO_4_) and the absorbance of each well was measured at 450 nm, using an ELISA plate reader. Duplicates of each sample were run. The antigen levels in sera were expressed as *A*_450 _values. *A*_450 _values of samples that were above 2.1 times of the negative controls were considered as positive [17].

Regression analysis was used to display the linearity and correlations. Analysis of data was performed using SPSS 13.0 (Chicago, IL, USA).

**Figure 1:**
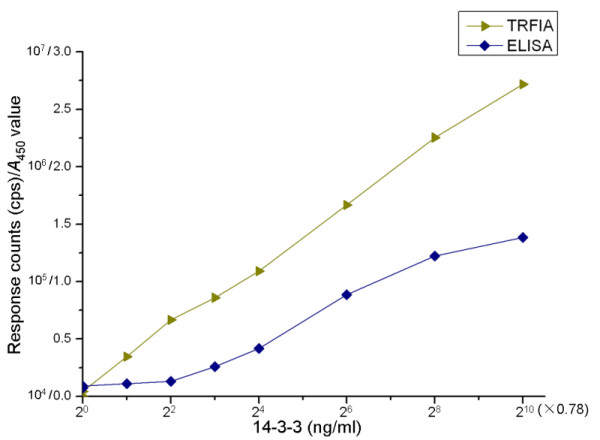
**Analytical performance of 14-3-3 calibrators by TRFIA and ELISA; y denotes response counts or *A***_**450 **_**values of calibrators; x indicates 14-3-3 concentration**.

The detection limit of TRFIA was defined by the fluorescence of the zero calibrator plus two SD, which was repeated 10 times in one experiment. According to the fluorescence count of the 14-3-3 zero calibrator in the TRFIA method, the detection limit of 14-3-3-TRFIA was 0.78 ng/ml. The calibration curve was linear from 0.78 - 800 (0.78 × 2^0 ^- 0.78 × 2^10^) ng/ml (Figure 1). The equation was:

for the calibration curve of this TRFIA, where y indicates values of the response counts (cps); x indicates concentration of calibrator (ng/ml).

The detection limit of 14-3-3-ELISA was 6.25 ng/ml, and its linear measurement range was from 6.25 to 200 (0.78 × 2^3 ^- 0.78 × 2^8^) ng/ml (Figure 1). The equation was:

for the calibration curve of 14-3-3-ELISA, where y indicates *A*_450 _values of the calibrators; x indicates concentration of calibrator (ng/ml) (The original data can be found in Additional file 1).

The calibration curve of 14-3-3-TRFIA showed a linear relationship over the concentration from 0.78-800 ng/ml. For 14-3-3-ELISA, the calibration curve was linear from 6.25-200 ng/ml before plateauing. All these data indicated that TRFIA has a better sensitivity and measurement range than ELISA.

The recovery rate was evaluated by comparing the measured and theoretical values. The recovery rates of 14-3-3-TRFIA and 14-3-3-ELISA were analyzed by adding recombinant 14-3-3 proteins at two different concentrations (20 and 100 ng/ml) to healthy rabbit serum from Group B. The mean recovery rate of TRFIA ranged from 92.1% to 115.5%, and the mean recovery rate of ELISA ranged from 94.1% to 105.5%.

The average intra- and inter- assay variations were calculated for the precision of the assays. Intra-assay variations of the two assays were determined by measuring the same two calibrators (20 and 100 ng/ml) for 10 times repeatedly in one experiment. The variation was checked by calculating the

and the applied acceptance criteria for immunoassays are % RSD < 20 [18]. The average intra-assay variations of TRFIA and ELISA were 8.9% and 5.9%, respectively. Inter-assay variations of the two assays were determined by measuring the same two calibrators (20 and 100 ng/ml) on five independent days. The average inter-assay variation of TRFIA and ELISA were 12.2% and 7.4%, respectively.

The Eu^3+^-labeled McAb 5C6 and the HRP labeled McAb 5C6 were stable for at least half a year at -20°C, and the results of the two methods with same reagents were reproducible over the same period.

**Figure 2:**
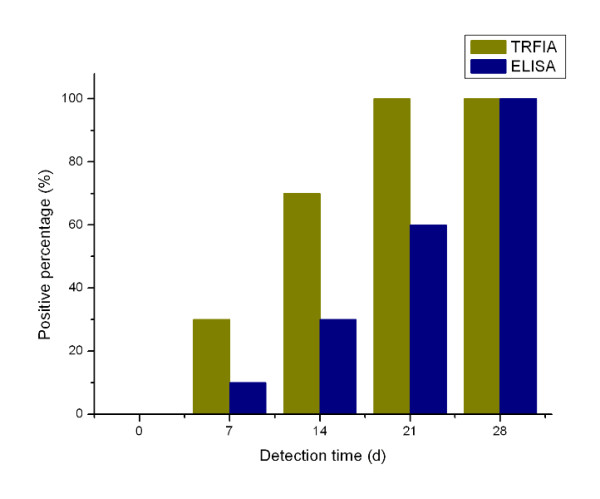
**Comparison of positive percentage of circulating antigen 14-3-3 detected by TRFIA and ELISA**.

Circulating antigen 14-3-3 protein in sera of rabbits collected at 0, 7, 14, 21, 28 days post-infection was measured by TRFIA and ELISA simultaneously, the positive percentages of TRFIA were 0, 30, 70, 100 and 100, respectively, and the positive percentages of ELISA were 0, 10, 30, 60 and 100 (Figure 2). The positive rate of TRFIA was significantly higher than that of ELISA within 21 days post-infection, but both assays reached 100% at 28 days post-infection (The original data can be found in Additional file 2 and 3).

There was a strong positive relationship between the worm burden, egg burden and the levels of 14-3-3 protein within individual rabbits (The original data can be found in Additional file 4), and it could be also found that levels of 14-3-3 protein in rabbits infected with 1500 cercariae of *S. japonicum *were higher than in those infected with 500 cercariae of *S. japonicum*. (The original data are not shown).

These results proved that the detection limit and the sensitivity of TRFIA are higher than those of ELISA, and TRFIA has stronger early diagnostic potential for schistosome infection. At present, the majority of schistosomiasis patients in epidemic areas of China have low infection levels. Circulating antigen levels are probably much lower than the detection limit of ELISA, indicating the need to develop more sensitive detection methods of schistosome circulating antigens [19].

TRFIA is based on the use of lanthanide chelate labels with unique fluorescence properties. Lanthanide chelate shows narrow and strong emission bands around 600 nm and an exceptionally long decay time, which allows the elimination of the high background of the fluorescent labels and increases the sensitivity and specificity of detection methods [20]. The sensitivity is further increased due to the dissociation-enhancement principle [21]. Eu^3+^-chelate is the most commonly used label in time resolved fluorometry-based analysis because of its higher fluorescence yield and lower background than other lanthanide complexes [14]. Therefore, TRFIA has the advantages of excellent sensitivity, wide measurement range and no radioactivity. A pair of monoclonal antibodies against signal transduction protein 14-3-3 of *S. japonicum *with highly specificity and affinity [12] was chosen to develop a sandwich TRFIA for detecting 14-3-3 protein. This assay combines characteristics of immunological methods and the sensitivity of a time-resolved fluorescence revelation system.

In this experiment, circulating antigen 14-3-3 protein in sera of rabbits collected post-infection was measured by TRFIA, and positive percentages of TRFIA could reach 100% after 21 days. Thus, for early diagnosis of *Schistosoma *infection, this TRFIA, combined with other detection methods [22,23], could obtain more satisfying results. The value of this TRFIA for clinical diagnosis of schistosomiasis japonica should also be further assessed.

## Competing interests

The authors declare that they have no competing interests.

## Authors' contributions

BH and CXY designed the study and interpreted the data; XRY, LJS, WZ and XDK collected the samples; JZ and JW did data analysis; CYQ performed the majority of experiments and prepared the manuscript. BH and CXY revised and finalized the manuscript. All authors read and approved the final manuscript.

## Supplementary Material

Additional file 1 **The original data of analytical performance of 14-3-3 calibrators by TRFIA and ELISA**. Click here for file

Additional file 2 **The original data of the detection results of 14-3-3 in sera of Group A and B measured by TRFIA**. Click here for file

Additional file 3 **The original data of the detection results of 14-3-3 in sera of Group A and B measured by ELISA**. Click here for file

Additional file 4 **Worm burden, egg burden and the levels of 14-3-3 protein within individual rabbits at 42 days post-infection**. Click here for file
