# Supplementary material for: Detection of the circulating antigen 14-3-3 protein of Schistosoma japonicum by time-resolved fluoroimmunoassay in rabbits
Source: Parasit Vectors. 2011 May 28;4:95. doi: 10.1186/1756-3305-4-95 (PMC3115898; doi:10.1186/1756-3305-4-95)
Supplement: Additional file 1 — The original data of analytical performance of 14-3-3 calibrators by TRFIA and ELISA. [file 1756-3305-4-95-S1.DOC]

**Table 1: The original data of analytical performance of 14-3-3 calibrators by TRFIA and ELISA**

|  | Calibrators  (ng/mL) | NC | 0 | 0.78 | 1.56 | 3.12 | 6.25 | 12.5 | 50 | 200 | 800 |
| --- | --- | --- | --- | --- | --- | --- | --- | --- | --- | --- | --- |
| TRFIA | response counts | 7180 | 5843 | **11001** | 22121 | 46213 | 72277 | 122800 | 463590 | 1795312 | 5202319 |
| ELISA | *A*_450_ value | 0.122 | 0.074 | 0.089 | 0.112 | 0.129 | **0.258** | 0.418 | 0.886 | 1.221 | 1.385 |
