# Supplementary material for: Detection of the circulating antigen 14-3-3 protein of Schistosoma japonicum by time-resolved fluoroimmunoassay in rabbits
Source: Parasit Vectors. 2011 May 28;4:95. doi: 10.1186/1756-3305-4-95 (PMC3115898; doi:10.1186/1756-3305-4-95)
Supplement: Additional file 2 — The original data of the detection results of 14-3-3 in sera of Group A and B measured by TRFIA. [file 1756-3305-4-95-S2.DOC]

**Table 2: The original data of the detection results of 14-3-3 in sera of Group A and B measured by TRFIA**

| No. | 0d | 7d | 14d | 21d | 28d |
| --- | --- | --- | --- | --- | --- |
| Group A1 | 5625 | 9856 | **16098** | **27259** | **32321** |
| Group A2 | 6351 | 8903 | 10365 | **19861** | **94687** |
| Group A3 | 7856 | 9581 | 11538 | **16921** | **31901** |
| Group A4 | 9379 | **15232** | **20068** | **43985** | **44687** |
| Group A5 | 6492 | 7455 | 8599 | **21033** | **33085** |
| Group A6 | 6084 | 9623 | **15867** | **36212** | **39308** |
| Group A7 | 5327 | 8526 | **15849** | **32054** | **52101** |
| Group A8 | 6376 | 9231 | **18421** | **30321** | **63238** |
| Group A9 | 9234 | **15117** | **17789** | **28562** | **40627** |
| Group A10 | 9077 | **15981** | **19548** | **43679** | **74673** |
| Positive percentage (%) | 0 | 30 | 70 | 100 | 100 |
| Group B1 | 5521 | 6172 | 8236 | 6921 | 7135 |
| Group B2 | 8561 | 6197 | 7769 | 8491 | 9386 |
| Positive percentage (%) | 0 | 0 | 0 | 0 | 0 |
