# Supplementary material for: Detection of the circulating antigen 14-3-3 protein of Schistosoma japonicum by time-resolved fluoroimmunoassay in rabbits
Source: Parasit Vectors. 2011 May 28;4:95. doi: 10.1186/1756-3305-4-95 (PMC3115898; doi:10.1186/1756-3305-4-95)
Supplement: Additional file 3 — The original data of the detection results of 14-3-3 in sera of Group A and B measured by ELISA. [file 1756-3305-4-95-S3.DOC]

**Table 3: The original data of the detection results of 14-3-3 in sera of Group A and B measured by ELISA**

| No. | 0d | 7d | 14d | 21d | 28d |
| --- | --- | --- | --- | --- | --- |
| Group A1 | 0.089 | 0.122 | 0.162 | 0.172 | **0.217** |
| Group A2 | 0.108 | 0.133 | 0.161 | **0.263** | **0.852** |
| Group A3 | 0.088 | 0.087 | 0.091 | **0.311** | **0.479** |
| Group A4 | 0.110 | **0.221** | **0.317** | **0.461** | **0.514** |
| Group A5 | 0.096 | 0.069 | 0.079 | 0.146 | **0.254** |
| Group A6 | 0.076 | 0.085 | 0.073 | 0.158 | **0.259** |
| Group A7 | 0.076 | 0.075 | 0.121 | **0.213** | **0.301** |
| Group A8 | 0.085 | 0.079 | 0.076 | 0.155 | **0.519** |
| Group A9 | 0.078 | 0.136 | **0.267** | **0.436** | **0.534** |
| Group A10 | 0.085 | 0.171 | **0.286** | **0.412** | **0.610** |
| Positive percentage (%) | 0 | 10 | 30 | 60 | 100 |
| Group B1 | 0.072 | 0.086 | 0.078 | 0.081 | 0.082 |
| Group B2 | 0.076 | 0.071 | 0.086 | 0.076 | 0.072 |
| Positive percentage (%) | 0 | 0 | 0 | 0 | 0 |
