# Supplementary material for: Detection of the circulating antigen 14-3-3 protein of Schistosoma japonicum by time-resolved fluoroimmunoassay in rabbits
Source: Parasit Vectors. 2011 May 28;4:95. doi: 10.1186/1756-3305-4-95 (PMC3115898; doi:10.1186/1756-3305-4-95)
Supplement: Additional file 4 — Worm burden, egg burden and the levels of 14-3-3 protein within individual rabbits at 42 days post-infection. [file 1756-3305-4-95-S4.DOC]

**Table 4: Worm burden, egg burden and the levels of 14-3-3 protein within individual rabbits at 42 days post-infection**

| No. | Worm burden | Liver egg burden  (×10^4^ /g) | TRFIA | ELISA |
| --- | --- | --- | --- | --- |
| Group A1 | 417 | 5.56 | 142324 | 1.338 |
| Group A2 | 375 | 3.95 | 101916 | 0.894 |
| Group A3 | 211 | 2.74 | 41901 | 0.523 |
| Group A4 | 247 | 2.99 | 54667 | 0.471 |
| Group A5 | 192 | 2.60 | 55085 | 0.505 |
| Group A6 | 269 | 3.12 | 66358 | 0.565 |
| Group A7 | 358 | 3.97 | 72191 | 0.797 |
| Group A8 | 322 | 3.86 | 83256 | 0.722 |
| Group A9 | 283 | 3.18 | 70629 | 0.566 |
| Group A10 | 405 | 4.92 | 144673 | 1.011 |
| Group B1 | 0 | 0 | 7923 | 0.077 |
| Group B2 | 0 | 0 | 8126 | 0.068 |
